# Supplementary material for: Key theories and technologies and implementation mechanism of parallel computing for ternary optical computer
Source: PLoS One. 2023 May 8;18(5):e0284700. doi: 10.1371/journal.pone.0284700 (PMC10166507; doi:10.1371/journal.pone.0284700)
Supplement: Attachment — Submitted filename: Response to Reviewers.docx [file pone.0284700.s001.docx]

List of Changes in Responses to Editors’ and Reviewers’ Comments on “Key Theories and Technologies and Implementation Mechanism of Parallel Computing for Ternary Optical Computer”

Dear Ph.D. Talib Al-Ameri,

On behalf of my co-authors, we thank you very much for all you have done on the manuscript entitled “Key Theories and Technologies and Implementation Mechanism of Parallel Computing for Ternary Optical Computer” (**ID: PONE-S-22-37459**). Meanwhile, we also appreciate you and reviewers very much for your helpful and constructive comments and suggestions on our manuscript. We have studied comments carefully and have made correction. The Revisions are marked in the paper. The uploaded please find the revised version. Should you have any questions please do not hesitate to let us know. We would like to express our great appreciation to you and the reviewers for the comments and suggestions on our paper.

Looking forward to hearing from you.

Best Regards,

Sulan ZHANG

Corresponding author:

Name: Sulan ZHANG

E-mail: zhangsl000111@163.com

List of Responses

Dear Ph.D. Talib Al-Ameri and Reviewers:

Thank you very much for your letter and the reviewers’ comments and suggestions concerning on our manuscript entitled “Key Theories and Technologies and Implementation Mechanism of Parallel Computing for Ternary Optical Computer” (**ID: PONE-S-22-37459**). Those comments and suggestions are all valuable and very helpful for revising and improving our paper, as well as the important guiding significance to our researches. We have studied comments carefully and have made corrections which we hope meet with approval. Revised portions are marked in blue in the Revised Manuscript. The main corrections in the paper and the responds to the reviewer’s comments are as follows:

1. **General revisions**

There are some modifications. List of Changes in the revised paper as follows:

1. In the 26^th^ line, TOC-----> the TOC
2. In the 29^th^ line, TOC-----> the TOC
3. The 30^th^ line was added.
4. In the 81^th^ line, TOP-----> Ternary Optical Processor (TOP)
5. In the 109^th^ line, TOP -----> the TOP
6. In the 116^th^ line, “Step 1:” was added.
7. In the 118^th^ line, “Step 2:” was added.
8. In the 120^th^ line, “Step 3:” was added.
9. In the 124^th^ line, the sentence “The calculation process of Parallel carry-free optical adders is divided into three steps as shown in Fig 2” and the Fig 2 were added.
10. In the 201-202^th^ line, the Fig 5 was added.
11. In the 238^th^ line, “Results and discussion” was added.
12. In the 324^th^ line, “C is the result of the operand a and the operand b.” was added.

More detailed modifications please refer to the Revised Manuscrip.

2. **Responds to the Reviewers**

**Reviewer #1:**

Reviewer #1: The paper introduced the related technologies to exert the ability of TOC parallel computing, and gave an experimental comparison. The scientific content and the results presented in the paper are adequate for publication.

**Response to Reviewer #1:**

I would like to thank the reviewer for his affirmation of this research

**Reviewer #2:**

Ternary Optical Computer (TOC) is more advanced than traditional computer systems in parallel computing. Based on huge amounts of repeated computations, this paper systematically elaborates the key theories and technologies of parallel computing for the TOC through a programming platform. And experiments are carried out to show the effectiveness of the present theories and technologies for parallel computing, as well as the feasibility of the implementation method of the programming platform. Based on the study of the TOC in this paper, more complex parallel computing can be realized in the future. The scientific content and the results presented in the paper are interesting and valuable. However, I have some suggestions as follows.

(1) In the second paragraph in the section 3.1, please give the full name of TOP.

(2) In section 3.3, the working process of parallel carry-free optical adder should be charted to show the details.

(3) Pay attention to writing details, for example: the second Paragraph in the section 3.3, in the sentence “Considering that TOP has three ...” , TOP should be the TOP.

The paper can be accepted for publication after the authors finish the above modifications.

First I would like to thank the reviewer for his affirmation of this research. The reply for the **reviewer #2 is as follows:**

**Question 1:** In the second paragraph in the section 3.1, please give the full name of TOP.

**Response to Reviewer #2:**

Thanks a lot for your suggestion. In 81th line , the full name of TOP was given, it is marked in blue.

**Question 2:** In section 3.3, the working process of parallel carry-free optical adder should be charted to show the details.

**Response to Reviewer #2:**

In 124th line , the working process of parallel carry-free optical adder should be charted to show the details as shown Fig 2.

**Question 3:** Pay attention to writing details, for example: the second Paragraph in the section 3.3, in the sentence “Considering that TOP has three ...” , TOP should be the TOP.

**Response to Reviewer #2:**

There are some modifications. List of Changes in the Revised Manuscript as follows:

1. In the 26^th^ line, TOC-----> the TOC
2. In the 29^th^ line, TOC-----> the TOC
3. The 30^th^ line was added.
4. In the 81^th^ line, TOP-----> Ternary Optical Processor (TOP)
5. In the 109^th^ line, TOP -----> the TOP
6. In the 116^th^ line, “Step 1:” was added.
7. In the 118^th^ line, “Step 2:” was added.
8. In the 120^th^ line, “Step 3:” was added.
9. In the 124^th^ line, the sentence “The calculation process of Parallel carry-free optical adders is divided into three steps as shown in Fig 2” and the Fig 2 were added.

More detailed modifications please refer to the Revised Manuscrip.
